# Unraveling the Links Between the Initiation of Ventilation and Brain Injury in Preterm Infants

**DOI:** 10.3389/fped.2015.00097

**Published:** 2015-11-10

**Authors:** Samantha K. Barton, Mary Tolcos, Suzie L. Miller, Charles C. Roehr, Georg M. Schmölzer, Peter G. Davis, Timothy J. M. Moss, Domenic A. LaRosa, Stuart B. Hooper, Graeme R. Polglase

**Affiliations:** ^1^The Ritchie Centre, Hudson Institute of Medical Research, Melbourne, VIC, Australia; ^2^Department of Obstetrics and Gynecology, Monash University, Melbourne, VIC, Australia; ^3^Newborn Services, John Radcliffe Hospital, Oxford University Hospitals, Oxford, UK; ^4^Department of Pediatrics, University of Alberta, Edmonton, AB, Canada; ^5^Centre for the Study of Asphyxia and Resuscitation, Neonatal Research Unit, Royal Alexandra Hospital, Alberta Health Services, Edmonton, AB, Canada; ^6^Neonatal Services, Newborn Research Centre, The Royal Women’s Hospital, Melbourne, VIC, Australia

**Keywords:** resuscitation, tidal volume, cerebral palsy, brain injury, premature, respiratory support, lungs

## Abstract

The initiation of ventilation in the delivery room is one of the most important but least controlled interventions a preterm infant will face. Tidal volumes (*V*_T_) used in the neonatal intensive care unit are carefully measured and adjusted. However, the *V*_T_s that an infant receives during resuscitation are usually unmonitored and highly variable. Inappropriate *V*_T_s delivered to preterm infants during respiratory support substantially increase the risk of injury and inflammation to the lungs and brain. These may cause cerebral blood flow instability and initiate a cerebral inflammatory cascade. The two pathways increase the risk of brain injury and potential life-long adverse neurodevelopmental outcomes. The employment of new technologies, including respiratory function monitors, can improve and guide the optimal delivery of *V*_T_s and reduce confounders, such as leak. Better respiratory support in the delivery room has the potential to improve both respiratory and neurological outcomes in this vulnerable population.

## Preterm Birth, Brain Injury, and Ventilation Requirement

Preterm birth, defined as birth prior to 37 completed weeks of gestation, affects 7–12% of births worldwide (1). Preterm babies have underdeveloped lungs, characterized by fewer alveoli, less surfactant, and a thicker blood–gas barrier that reduces oxygen and carbon dioxide diffusion into the bloodstream (2, 3). Many of these babies have impaired lung function, rendering them unable to survive without assistance. In Australia, 28% of all infants required some form of respiratory support in the delivery room, with suction and oxygen support encompassing the majority of this support. However, 7.5% required intermittent positive pressure ventilation (IPPV); the requirement for IPPV increases with decreasing gestational age (4). Respiratory support is the cornerstone of successful neonatal resuscitation (5); it allows appropriate transition from fetal to neonatal circulation as well as aiding in lung liquid clearance and functional residual capacity establishment. However, it is now well established that assisted ventilation increases inflammation and injury to the preterm lungs (6).

The effect of respiratory support at birth on other organ systems, especially the brain, remains relatively unexplored. This is of particular importance given that preterm infants have a significantly increased risk of acute and chronic brain injury compared to term infants. Children born preterm have higher rates of sensory deficits, learning disabilities, and cerebral palsy than children born at term (7). Compared to term infants, babies born extremely preterm have a greater incidence of moderate–severe disability (20.3 vs. 2.5%), moderate–severe developmental delay (16 vs. 2%) and cerebral palsy (9.8 vs. 0%) at 2 years of age (8). Given the higher incidence of poor neurodevelopmental outcomes in preterm infants, reducing the incidence and severity of brain injury in this population is essential to enhance the long-term health and welfare of individuals born preterm.

There is increasing evidence from animal studies that ventilation-induced lung injury (VILI) leads to systemic (9–11) and brain (12, 13) inflammation and injury. Furthermore, inflammatory and hemodynamic pathways play a critical role in the pathogenesis of brain injury in preterm infants (14–16). Thus, given the underlying risk of brain injury in preterm babies, the requirement for ventilation further exacerbates the probability of acute injury and chronic disability suggesting injurious ventilation to be an important contributor to brain damage in the preterm infant. It is therefore imperative that a baby born preterm receives the safest possible respiratory support in the delivery room.

## Initiation of Ventilation in the Delivery Room

Neonatologists are familiar with the concept of VILI and are increasingly careful in the neonatal intensive care unit (NICU) to apply mechanical ventilation strategies that are gentle and minimize trauma to the lungs (17, 18). Although ventilation and supplemental oxygen therapy are two of the most common interventions used in the NICU, neonatologists appear less aware that the same gentle approach should be applied to reduce VILI in the delivery room (17, 18). All modern ventilators allow adequate monitoring followed by continuous adjustment of setting (19) to achieve gentle ventilation, yet the same strategies have not been employed in the delivery room (20–23). The lack of adequate monitoring in the delivery room may influence the development of respiratory distress syndrome and bronchopulmonary dysplasia (BPD).

### Devices for Respiratory Support in the Delivery Room – Accuracy and Efficacy

The International Liaison Committee on Resuscitation (ILCOR) advises on the techniques and the equipment used for neonatal resuscitation (24). Acknowledging the scant evidence available regarding the optimal initial airway management of preterm infants, ILCOR has summarized the most significant knowledge gaps and research priorities regarding neonatal resuscitation as (i) “the optimal ventilatory strategy for neonatal resuscitation in the delivery room”, (ii) “airway pressures, inspiratory times, devices, timing, and volumes in relation to gestational age”, and (iii) “options for providing feedback to rescuers to ensure correct ventilation rates and tidal volumes” (25). The application of safe and effective manual ventilation in the delivery room relies on several components – the safety and reliability of the equipment and the operator’s skills and clinical expertise when handling it.

According to recent surveys, the most commonly used devices for the initial respiratory support of newborn infants are self-inflating bags, flow-inflating (anesthetic) bags, and T-piece resuscitators (26–29). The delivered peak inspiratory pressure (PIP) and *V*_T_ are highly device dependent (30–32). A fundamental difference between T-piece resuscitators and self-inflating bags is that the T-piece resuscitators have pressure-limiting valves for PIP and positive-end expiratory pressure (PEEP), whereas self-inflating bags do not. Pressure manometers for self-inflating bags are available but rely on the operator to watch the manometer and adjust their technique to achieve the desired pressure. PEEP valves are an optional addition of doubtful clinical effectiveness (33). Therefore, self-inflating bags are often used without pressure manometers (26), despite evidence showing a pressure manometer significantly reduces the median applied PIP (34). T-piece resuscitators provide accurate, reliable, well-controlled PIP and PEEP compared to self-inflating bags (34, 35).

Pressure and volume delivery depends on a secure patient–device interface. Initial positive pressure ventilation is generally applied via a face mask. However, mask leaks are frequently encountered due to an inadequate mask seal around the infants’ nose and mouth. This can inadvertently lead to variable *V*_T_ delivery (36, 37). Fluctuating mask leak may lead to either inadequate ventilation or dangerously high *V*_T_s being delivered. Furthermore, airway obstruction is also a significant problem in the initial respiratory support, and also results in inadequate ventilation. The problems of leak and airway obstruction highlight the need for monitoring of both PIP and *V*_T_ during ventilation in the delivery room; this would aid clinicians in accurate placement of the face mask to minimize mask leak, and the repositioning of the head and neck in the case of airway obstruction, thus optimizing ventilation and prevenient delivery of excessive *V*_T_s. Training of the correct mask hold technique and of manual ventilation has been shown to improve the ventilation of neonates (38).

Significant improvements in the consistency of *V*_T_, rate, and rhythm of neonatal resuscitation have been demonstrated by the use of respiratory function monitors and auditory prompts (29, 39–41). The use of respiratory function monitors in clinical practice is not standard, but studies investigating their use during neonatal resuscitation are underway. Measuring exhaled CO_2_ also shows promise as an indicator of adequate lung aeration with correlations drawn between exhaled CO_2_ and end lung volume in animal models (42); this could be beneficial in the delivery room by representing an established functional residual capacity. Mian et al. found that the use of a flow sensor during ventilation within the delivery room allowed the monitoring of *V*_T_ and exhaled CO_2_, though the study was limited to babies receiving continuous positive airway pressure (CPAP) (43). The implementation of respiratory function monitors and/or flow sensors may, therefore, provide additional technical support for clinicians in ensuring correct ventilation of neonates within the delivery room. This is particularly relevant given that the younger and sicker preterm babies, that are more inclined to require respiratory support, are also more likely to be delivered in tertiary centers that are able to employ such devices.

In summary, the ability of any resuscitation device to deliver accurate pressures and *V*_T_s is dependent on both the device and the ability of the operator to use it skillfully. It is pertinent to note that none of the currently available devices provide clinicians with any feedback of the *V*_T_ delivered, a critical omission given the potential for significant lung injury.

### High Tidal Volume Ventilation in the Delivery Room: Lung and Systemic Consequences

The *V*_T_ delivered during neonatal resuscitation is not well controlled; given that volume distension of the lungs rather than pressure or oxygen toxicity is the important factor causing the initiation of injury (17, 44–49), better *V*_T_ monitoring may improve neonatal outcomes. Devices used in the delivery room allow for the delivery of consistent PIP, but the *V*_T_ achieved will vary not only due variable leak, but also according to the changing compliance and resistance of the lung, the stiffness of the chest wall, and the volume of lung liquid retained within the airways (50). Trials at the Royal Women’s Hospital, Melbourne, Australia found the *V*_T_ delivered to preterm infants varied from 0 to >30 mL/kg when the PIP was set at 30 cmH_2_O (50). Importantly, 85% of these infants inadvertently received a *V*_T_ higher than recommended (45, 51), which is likely to be injurious. This study also demonstrated the difficulty clinicians face in assessing *V*_T_ in the absence of appropriate feedback from the devices; five resuscitators in the delivery room could not estimate the *V*_T_, one over-estimated and 14 under-estimated the actual *V*_T_ delivered (50). These studies clearly illustrate that preterm infants are inadvertently receiving high *V*_T_ in the delivery room.

The links between high *V*_T_ ventilation and VILI are well established. Studies in preterm lambs highlighted that ventilation, regardless of the strategy used, triggers an inflammatory response in the lung (6). However, lung inflammation and injury are amplified when lambs received a high *V*_T_ (10 mL/kg) compared to a normal *V*_T_ (5 mL/kg) (52). Indeed, as few as three large *V*_T_ breaths is sufficient to initiate an inflammatory response in the lungs (53, 54), resulting in lung inflammation and injury leading to BPD. The maintenance of high *V*_T_ for up to 15 min leads to profound lung inflammation and injury (55, 56). Further, VILI can trigger a systemic inflammatory response (9, 10), which can cause inflammation in multiple organs (10, 57). Term and late preterm infants have an acute systemic inflammatory response after 2 h of ventilation evidenced by increased plasma pro-inflammatory cytokines interleukin (IL)-8 (2.5-fold), IL-1β (7.5-fold), and TNF-α (10-fold) and a decrease in the anti-inflammatory cytokine IL-10 (by 90%) (58). The initiation of a systemic inflammatory cascade is a known mechanism of cerebral white matter inflammation and injury (59). Strong associations have been made between VILI and BPD (60), and BPD and cerebral palsy (61), and the duration of ventilation increases the risk of white matter pathology (62). Together these studies suggest a causal link between ventilation, an inflammatory cascade and brain pathology. However, until recently, the effects of the initial resuscitation in the delivery room on the preterm brain were unknown.

### High Tidal Volume Ventilation in the Delivery Room: Consequences for the Preterm Brain

**Figure 1:**
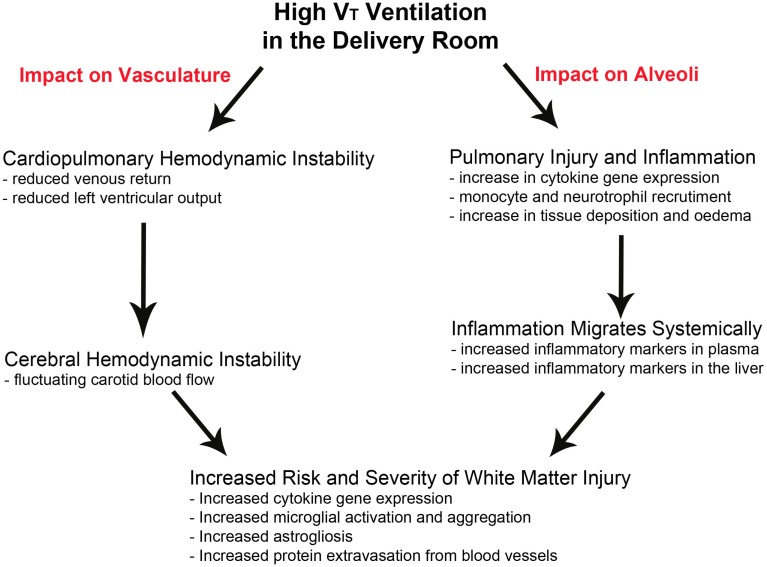
**Pathways leading to ventilation-induced brain inflammation and injury**. The pathways involved in the progression of brain injury following high *V*_T_ ventilation in the delivery room. High *V*_T_ ventilation can lead to cardiopulmonary hemodynamic instability leading to variable and fluctuating carotid blood flow while also instigating an inflammatory response in the lungs, which can migrate systemically. Both pathways can occur independently or together to increase the risk and severity of cerebral white matter injury.

Recent studies in preterm lambs (13, 63), coupled with studies in preterm infants (64), have demonstrated that the initiation of ventilation causes brain pathology through the same two mechanistic pathways key to perinatal brain injury, hemodynamic instability, and a localized cerebral inflammatory response (13, 14) (Figure 1).

Over-distension of the preterm lungs as a result of high *V*_T_ or end-expiratory lung volume within the first minutes to hours after birth, compresses the alveolar capillaries leading to pulmonary hemodynamic instability. This, in turn, alters pulmonary venous return and cardiac output and results in large swings in cerebral blood flow (CBF) (13, 65–67). Similarly, the application of IPPV and PEEP induces variability in intrathoracic pressure, which can independently alter cardiac function by affecting preload, afterload, heart rate, and myocardial contractility (68), which will also influence cerebral hemodynamics. This is not usually a problem in normal term infants, as they have the ability to maintain near-constant blood flow in the face of changing perfusion pressures by altering cerebral vasculature resistance (autoregulation) (69). However, it is now clear that preterm infants <30 weeks have episodes of impaired autoregulation, primarily due to their cerebral immaturity (70). Episodes of impaired autoregulation are reported to occur as much as 50% of the time during the first 5 days of life (71). Without intact autoregulation, abnormal CBF can cause hypoxia/ischemia (if CBF is low) or cerebral hemorrhage (if CBF is high or rapidly fluctuating between low and high flows).

Over-distension of the alveoli also initiates a pulmonary inflammatory response, which migrates systemically to the brain before crossing the blood–brain barrier (72) and activating a localized inflammatory response (13). This results in a profound increase in pro-inflammatory cytokine gene expression in the brains of ventilated preterm lambs (73). The underlying mechanism of perinatal white matter injury is upregulation of proinflammatory cytokines and diffuse activation of microglia within the immature white matter (59). The microglia mediate the local response by generating free radicals and amplifying cytokine production, which are important causes of brain injury. Increased pro-inflammatory cytokines (IL-1β, IL-6, and TNF-α) can also compromise the cerebral vasculature reducing its ability to protect against abnormal CBF, as well as reducing the integrity of the blood–brain barrier making it more prone to hemorrhage (74, 75). Thus, the inflammatory cascade may injure the preterm brain via direct gliosis-induced toxicity or through increased blood–brain barrier permeability, as well as via perturbation of the cerebral vasculature leading to abnormal CBF.

**Figure 2:**
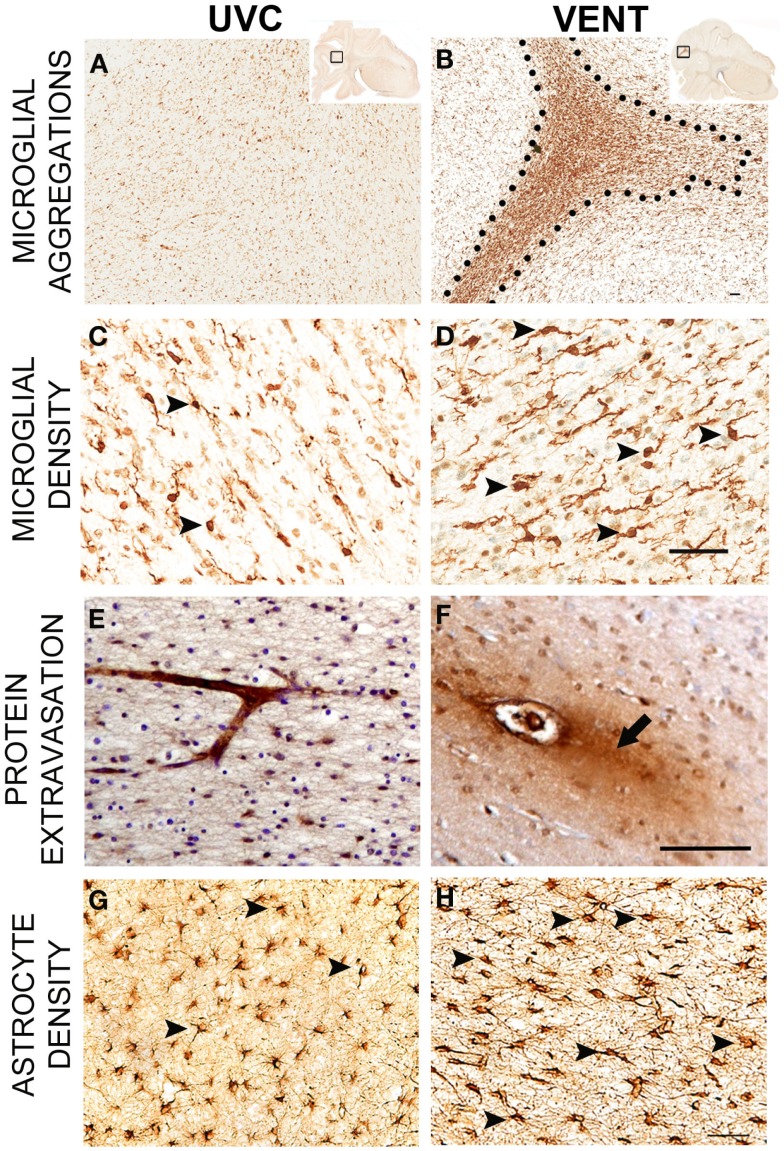
**Pathology of ventilation-induced brain inflammation and injury**. Comparative immunohistological findings from the subcortical white matter of the parietal lobe from an unventilated control lamb (UVC; left panel) and a lamb ventilated with a high *V*_T_ (Vent; right panel). Lambs ventilated with a high *V*_T_ have larger, denser microglial aggregations **(A,B)**, increased microglial density [**(C,D)**; arrowheads indicate Iba-1 positive microglia], increased blood–brain barrier permeability evidenced by protein extravasation [**(E,F)**; arrow indicates protein extravasation from the vessel labeled with an anti-sheep serum antibody] as well as increased astrogliosis [**(G,H)**; arrowheads indicate GFAP-positive astrocytes]. Scale bar represents 50 μm. Images adapted from Ref. (73, 76).

High *V*_T_ ventilation produces a distinct pattern of pathology within the white matter (Figure 2). At a histological level, the inflammatory response is evident within the brain parenchyma with an increase in size and density of microglial aggregations; importantly these can be observed after only 2 h of ventilation (73, 76). Subsequent increases in markers of astrogliosis, oxidative stress, and cell death are also characteristics (13, 73, 76). Indicators of brain inflammation and injury are also evident using clinical tools. Using magnetic resonance spectroscopy (MRS), Skiöld et al. found higher concentrations of markers of neuronal damage and cell membrane turnover in lambs that received high *V*_T_ for the first 15 min compared to lambs that received a normal *V*_T_ (12). Using near-infrared spectroscopy (NIRS), Polglase et al. found highly variable cerebral oxygenation in preterm lambs ventilated with high *V*_T_, compared to lambs ventilated with a protective strategy (13). This has been mirrored clinically with low cerebral oxygenation in the delivery room during neonatal transition, measured using NIRS, being associated with IVH development (77). Permeability of the blood–brain barrier is also increased by high *V*_T_ ventilation (13, 76), this can have devastating consequences for the preterm infant as it allows passage of inflammatory mediators into the parenchyma (78).

The impact of high *V*_T_ ventilation in the delivery room on the preterm brain has now been assessed. Two groups of preterm infants ventilated with a *V*_T_ < 5.8 mL/kg and >5.8 mL/kg were assessed; 48% of the cohort analyzed were not intubated in the delivery room and of this proportion of babies, mask leak was monitored (with a mask leak >30% excluded from analysis). It was found that 51% (25/49) of infants receiving high *V*_T_ in the delivery room were diagnosed with IVH compared to only 13% (2/16) of infants receiving the lower *V*_T_. Furthermore, of the 25 infants who received the high *V*_T_ and developed IVH, 36% had the severest grade IV IVH (64). It is worth highlighting that, given the trial design, the babies requiring high *V*_T_ ventilation could have been more compromised than their low *V*_T_ ventilation counterparts rendering them more susceptible to lung and brain injury. Yet, despite this being a preliminary study, and groups being non-randomized, it highlights the requirement for further investigation into the critical relationship between high *V*_T_ ventilation in the delivery room and the potential for IVH in preterm babies.

Taken together, these studies highlight the intimate association between the lungs, heart, and brain during the initial resuscitation, and the critical need to consider the potential downstream consequences. Given that the pathways leading to brain damage can be activated as early as the first breaths delivered to a preterm infant in the delivery room, it is imperative that careful ventilation strategies, including *V*_T_ monitoring (18), as already practiced in most NICUs, are used in the delivery room. Indeed, studies have demonstrated that a lower *V*_T_ improves cerebral hemodynamic stability and reduces the inflammatory response. Polglase et al. demonstrated that a protective ventilation strategy, encompassing prophylactic surfactant, a sustained inflation (SI) and low *V*_T_, largely prevented molecular and histological brain injury compared to a high *V*_T_ (13) and also reduced signs of brain injury using MRS (12). Mian et al. supported this contention in a study of human preterm infants whereby neonates receiving *V*_T_ < 5.8 mL/kg had reduced rates of IVH than neonates receiving *V*_T_ > 5.8 mL/kg (64). These studies highlight the importance of controlling the *V*_T_ in the delivery room.

### The Delivery Room and Beyond: Strategies for a Continuum of Non-Invasive Respiratory Support

We have so far focused on the importance of monitoring respiratory parameters within the delivery room, with the most emphasis placed on *V*_T_, yet we must also highlight the increasing popularity of non-invasive respiratory support both in the delivery room and the NICU. The two most common forms of non-invasive respiratory support in the NICU are CPAP and nasal high flow therapy (nasal HFT). Non-invasive respiratory support is preferable over intubation and mechanical ventilation, given the prevention of adverse effects that can result from intubation (79). While CPAP is now routinely used in the delivery room, nasal HFT remains limited to the NICU.

A recent survey of delivery room stabilization practice of very preterm infants showed 77% of tertiary units used CPAP (80). While Singh and Oddie highlight the increased use of non-invasive respiratory support in the delivery room, they also acknowledge that there is a marked variation in practice between units (80). Further, the optimal timing of CPAP, as well as ways to deliver surfactant, remains elusive. The COIN trial randomized 610 extremely preterm infants to either CPAP in the delivery room, or intubation and mechanical ventilation, and found CPAP resulted in reduced need for supplementary oxygen at 28 days as well as reduced median days requiring ventilatory support (81), although CPAP was associated with a higher rate of pneumothoraces. Similar findings were demonstrated in the SUPPORT trial. Infants who received CPAP treatment less frequently required intubation, postnatal corticosteroids, required fewer days of mechanical ventilation and had better outcomes at 7 days (82) compared to infants that received mechanical ventilation and early surfactant therapy. There appears to be an apparent respiratory benefit at least in the short term, and this is well documented (83–85), maintaining respiratory benefits in the long term (86). However, despite these initial improvements, the use of CPAP in the delivery room has not translated into improved long-term neurological outcomes of preterm infants (87) with death or neurodevelopmental impairment at 18–22 months corrected age occurring in ~30% of infants in both groups. In animal models: CPAP did not reduce lung or systemic markers of inflammation after 3 h compared to preterm lambs receiving conventional mechanical ventilation (9). Given the pathways of ventilation-induced brain injury, these data can be extrapolated to allow the assumption that CPAP would not have protected the brains of these lambs from ventilation-induced inflammation. In the preterm brain, the advantages of early CPAP over delayed CPAP have been shown in a preterm baboon model (88), albeit “early CPAP” in this model commenced 24 h after delivery. A lamb model of early CPAP allowing prolonged ventilation has now been developed which successfully transitioned to nCPAP from nasal intermittent positive pressure ventilation (NIPPV) at 28 ± 11 min (89); further progress with a similar model will hopefully allow more in-depth assessment of the impact of early CPAP on the preterm brain.

It is worth also addressing the use of SIs in the delivery room, particularly their combination with CPAP, given their rapid introduction into conventional respiratory care. Extensive animal models have demonstrated the physiological advantages of SI in improving the cardiovascular transition at birth, establishing a functional residual capacity as well as assisting in uniform lung aeration (90, 91). Yet, the use of an SI does not correlate to reduced lung inflammation and injury (92). Furthermore, there is concern about the rapid increase in cardiopulmonary and cerebral hemodynamics after an SI. A recent study in term asphyxic lambs demonstrated that the more rapid increase in CBF after a 30-s SI resulted in increased vascular extravasation; a surrogate for IVH (93). The translation to human studies has to date demonstrated variable results. An SI has been associated with increased incidence of patent ductus arteriosus and a trend for increased incidence of IVH (94, 95). The use of an SI prior to nCPAP in the delivery room appears to improve ventilation parameters and reduce the time on mechanical ventilation but there was also a trend towards higher rates of pneumothoraces (96, 97). The observed differences between the animal and clinical studies may be due to the actual delivery of the SI – the animal studies were all conducted in intubated animals while clinical trials are delivering the SI largely using facemasks. Therefore, the difference in the interface may alter the efficacy of the delivery of the SI. More studies are required to determine the efficacy of a SI for use in the DR before this becomes standard care.

Nasal HFT is becoming increasingly common as an alternative for CPAP, but to date is mainly limited to use in the NICU. It allows the delivery of humidified blended air and oxygen via high flow nasal cannulae (HFNC) and can deliver a PEEP, such as CPAP (98). To date, studies have demonstrated similar effectiveness of CPAP and HFNC as post-extubation therapy, although HFNC was not quite as effective as CPAP in preventing extubation failure (99–101). Thus, HFNC appears a potential alternative mode of non-invasive ventilation for many preterm infants. Its ease of use, popularity with nurses and parents, and reduced rates of nasal trauma (102, 103) have resulted in rapid uptake of HFNC in neonatal units worldwide. Furthermore, a recent pilot study suggests that HFNC may be as effective as NIPPV in preventing endotracheal ventilation in the primary treatment of respiratory distress syndrome in premature infants <35 weeks (104). However, to date, no studies have determined the safety or efficacy of using HFNC in the delivery room as an alternative to CPAP or IPPV, nor have any long-term neurological outcomes of the trials been presented. Thus, HFNC remains a relatively untested, but potentially promising, future therapy for preterm respiratory support in the delivery room.

## Summary

Many preterm infants require IPPV in the delivery room. Due to the lack of sophistication of devices used to provide respiratory support to preterm infants in the delivery room, many of these babies receive inadvertently high *V*_T_, which can injure their immature lungs and brain. The safety and effectiveness of mask ventilation may be improved if respiratory function monitoring is used to help clinicians deliver appropriate *V*_T_s. Non-invasive respiratory support in the delivery room reduces the risks associated with endotracheal intubation. However, increased use of CPAP has not translated into improved long-term neurological outcomes for preterm infants. Many infants initially managed with CPAP eventually require intubation and mechanical ventilation increasing the potential for lung and brain injury. There is a critical need to improve respiratory care in the delivery room and minimize the number of babies receiving inadvertently injurious ventilation, thus reducing the risk and severity of adverse pulmonary and neurological outcomes.

## Author Contributions

SB, CR, GS, and GP all contributed to the conception and design of the review and SB, CR, GS, GP, MT, SM, PD, TM, DL and SH all contributed to the drafting, revising, and final approval of the version to be published.

## Conflict of Interest Statement

The authors declare that the research was conducted in the absence of any commercial or financial relationships that could be construed as a potential conflict of interest.

## Abbreviations

BPD, bronchopulmonary dysplasia; CBF, cerebral blood flow; IL, interleukin; IVH, intraventricular hemorrhage; NICU, neonatal intensive care unit; PEEP, positive end-expiratory pressure; PIP, peak inspiratory pressure; VILI, ventilation-induced lung injury; *V*_T_, tidal volume; WM, white matter.

## References

[1] Howson CP Kinney MV Lawn JE editors. March of Dimes, PMNCH, Save the Children, WHO. Born Too Soon: The Global Action Report on Preterm Birth. Geneva: World Health Organization (2012).

[2] Moss TJM. Respiratory consequences of preterm birth. Clin Exp Pharmacol Physiol (2006) 33(3):280–4. 10.1111/j.1440-1681.2006.04359.x 16487275

[3] Hillman N Kallapur S Pillow J Nitsos I Polglase G Ikegami M Inhibitors of inflammation and endogenous surfactant pool size as modulators of lung injury with initiation of ventilation in preterm sheep. Respir Res (2010) 11:151. 10.1186/1465-9921-11-151 21034485 PMC2978154

[4] Li Z Zeki R Hilder L Sullivan EA Australia’s mothers and babies 2011. Perinatal statistics series no. 28. Cat. no. PER 59. Canberra: AIHW National Perinatal Epidemiology and Statistics Unit (2013). p. 80–2.

[5] Kattwinkel J Newborn Life Support – Resuscitation at Birth. 5th ed American Academy of Pediatrics and American Heart Association Publication (2006).

[6] Jobe AH Hillman N Polglase G Kramer BW Kallapur S Pillow J. Injury and inflammation from resuscitation of the preterm infant. Neonatology (2008) 94(3):190–6. 10.1159/000143721 18832854

[7] Beck S Wojdyla D Say L Betran AP Merialdi M Requejo JH The worldwide incidence of preterm birth: a systematic review of maternal mortality and morbidity. Bull World Health Organ (2010) 88(1):31–8. 10.2471/BLT.08.062554 20428351 PMC2802437

[8] Doyle LW Roberts G Anderson PJ Outcomes at age 2 years of infants < 28 weeks’ gestational age born in Victoria in 2005. J Pediatr (2010) 156(1):49–53e1. 10.1016/j.jpeds.2009.07.013 19783004

[9] Polglase GR Hillman NH Ball MK Kramer BW Kallapur SG Jobe AH Lung and systemic inflammation in preterm lambs on continuous positive airway pressure or conventional ventilation. Pediatr Res (2009) 65(1):67–71. 10.1203/PDR.0b013e318189487e 18704000

[10] Hillman NH Moss TJM Kallapur SG Bachurski C Pillow JJ Polglase GR Brief, large tidal volume ventilation initiates lung injury and a systemic response in fetal sheep. Am J Respir Crit Care Med (2007) 176(6):575–81. 10.1164/rccm.200701-051OC 17641159 PMC1994225

[11] Tremblay LN Slutsky AS Ventilator-induced lung injury: from the bench to the bedside. Intensive Care Med (2006) 32(1):24–33. 10.1007/s00134-005-2817-8 16231069

[12] Skiöld B Wu Q Hooper SB Davis PG McIntyre R Tolcos M Early detection of ventilation-induced brain injury using magnetic resonance spectroscopy and diffusion tensor imaging: an *in vivo* study in preterm lambs. PLoS One (2014) 9(4):e95804. 10.1371/journal.pone.0095804 24759765 PMC3997476

[13] Polglase G Miller SL Barton SK Baburamani AA Wong FY Aridas JDS Initiation of resuscitation with high tidal volumes causes cerebral hemodynamic disturbance, brain inflammation and injury in preterm lambs. PLoS One (2012) 7(6):e39535. 10.1371/journal.pone.0039535 22761816 PMC3382197

[14] Polglase GR Miller SL Barton SK Kluckow M Gill AW Hooper SB Respiratory support for premature neonates in the delivery room: effects on cardiovascular function and the development of brain injury. Pediatr Res (2014) 75:682–8. 10.1038/pr.2014.40 24614803

[15] Barton SK Moss TJ Hooper SB Crossley KJ Gill AW Kluckow M Protective ventilation of preterm lambs exposed to acute chorioamnionitis does not reduce ventilation-induced lung or brain injury. PLoS One (2014) 9(11):e112402. 10.1371/journal.pone.0112402 25379714 PMC4224447

[16] Perlman JM McMenamin JB Volpe JJ. Fluctuating cerebral blood-flow velocity in respiratory-distress syndrome. Relation to the development of intraventricular hemorrhage. N Engl J Med (1983) 309:204–9. 10.1056/NEJM198307283090402 6866033

[17] Schmölzer GM Te Pas AB Davis PG Morley CJ Reducing lung injury during neonatal resuscitation of preterm infants. J Pediatr (2008) 153(6):741–5. 10.1016/j.jpeds.2008.08.016 19014815

[18] Wheeler K Klingenberg C Morley CJ Davis PG. Volume-targeted versus pressure-limited ventilation for preterm infants: a systematic review and meta-analysis. Neonatology (2011) 100(3):219–27. 10.1159/000326080 21701210

[19] Morley CJ Keszler M Ventilators do not breathe. Arch Dis Child Fetal Neonatal Ed (2012) 97:F392–4. 23080476 10.1136/fetalneonatal-2012-302137

[20] Milner A Murthy V Bhat P Fox G Campbell ME Milner AD Evaluation of respiratory function monitoring at the resuscitation of prematurely born infants. Eur J Pediatr (2015) 174(2):205–8. 10.1007/s00431-014-2379-2 25029987

[21] Schilleman K Siew ML Lopriore E Morley CJ Walther FJ te Pas A Auditing resuscitation of preterm infants by recording video and physiological parameters. Resuscitation (2012) 83:1135–9. 10.1016/j.resuscitation.2012.01.036 22322286

[22] Vento M Aguar M Leone TA Finer NN Gimeno A Rich W Using intensive care technology in the delivery room: a new concept for the resuscitation of extremely preterm neonates. Pediatrics (2008) 122(5):1113–6. 10.1542/peds.2008-1422 18977992

[23] Schmölzer GM Olischar M Raith W Resch B Reiterer F Müller W Delivery room resuscitation. Monatsschr Kinderheilkd (2010) 158:471–6.

[24] Nolan JP Soar J Zideman DA Biarent D Bossaert LL Deakin CD European resuscitation council guidelines for resuscitation 2010. Resuscitation (2010) 81:1219–76. 10.1016/j.resuscitation.2010.08.021 20956052

[25] Gazmuri RJ Nadkarni VM Nolan JP Arntz HR Billi JE Bossaert L Scientific knowledge gaps and clinical research priorities for cardiopulmonary resuscitation and emergency cardiovascular care identified during the 2005 International Consensus Conference on ECC and CPR science with treatment recommendations: a consensus statement from the International Liaison Committee on Resuscitation (American Heart Association, Australian Resuscitation Council, European Resuscitation Council, Heart and Stroke Foundation of Canada, InterAmerican Heart Foundation, Resuscitation Council of Southern Africa, and the New Zealand Resuscitation Council); the American Heart Association Emergency Cardiovascular Care Committee; the Stroke Council; and the Cardiovascular Nursing Council. Circulation (2007) 116(21):2501–12. 17993477 10.1161/CIRCULATIONAHA.107.186228

[26] O’Donnell CPF Davis PG Morley CJ. Positive pressure ventilation at neonatal resuscitation: review of equipment and international survey of practice. Acta Paediatr (2004) 93:583–8. 10.1111/j.1651-2227.2004.tb02981.x 15174776

[27] O’Donnell CPF Davis PG Lau R Dargaville PA Doyle LW Morley CJ. Neonatal resuscitation 2: an evaluation of manual ventilation devices and face masks. Arch Dis Child Fetal Neonatal Ed (2005) 90:F392–6. 10.1136/adc.2004.064709 15871989 PMC1721950

[28] Roehr CC Gröbe S Rüdiger M Hummler H Nelle M Proquitté H Delivery room management of very low birth weight infants in Germany, Austria and Switzerland – a comparison of protocols. Eur J Med Res (2010) 15:493–503. 10.1186/2047-783X-15-11-493 21159574 PMC3352658

[29] Mileder LP Urlesberger B Schwindt J Simma B Schmölzer GM. Compliance with guidelines recommending the use of simulation for neonatal and infant resuscitation training in Austria. Klin Padiatr (2014) 226(1):24–8. 10.1055/s-0033-1361106 24435789

[30] Bennett S Finer NN Rich W Vaucher YE. A comparison of three neonatal resuscitation devices. Resuscitation (2005) 67(1):113–8. 10.1016/j.resuscitation.2005.02.016 16081202

[31] Roehr CC Kelm M Fischer HS Bührer C Schmalisch G Proquitté H. Manual ventilation devices in neonatal resuscitation: tidal volume and positive pressure-provision. Resuscitation (2010) 81:202–5. 10.1016/j.resuscitation.2009.10.008 19926383

[32] Thio M Dawson JA Moss TJ Galinsky R Rafferty A Hooper SB Self-inflating bags versus T-piece resuscitator to deliver sustained inflations in a preterm lamb model. Arch Dis Child Fetal Neonatal Ed (2014) 99(4):F274–7. 10.1136/archdischild-2013-305239 24646620

[33] Morley CJ Dawson JA Stewart MJ Hussain F Davis PG The effect of a peep value on a laerdal neonatal self-inflating resuscitation bag. J Paediatr Child Health (2010) 46:51–6. 10.1111/j.1440-1754.2009.01617.x 19943861

[34] Hartung JC Dold SK Thio M Te Pas AB Schmalisch G Roehr CC. Time to adjust to changes in ventilation settings varies significantly between different T-piece resuscitators, self-inflating bags and manometer equipped self-inflating bags. Am J Perinatol (2014) 31:505–12. 10.1055/s-0033-1354562 24000108

[35] Roegholt E van Vonderen JJ Walther FJ Roehr CC Te Pas AB. Do we deliver the pressures we intend to when using a T-piece resuscitator? PLoS One (2013) 8:e64706. 10.1371/journal.pone.0064706 23717652 PMC3661533

[36] Wood FE Morley CJ Dawson JA Kamlin CO Owen LS Donath S Assessing the effectiveness of two round neonatal resuscitation masks: study 1. Arch Dis Child Fetal Neonatal Ed (2008) 93:F235–7. 10.1136/adc.2007.117713 18039749

[37] Wood FE Morley CJ Dawson JA Kamlin CO Owen LS Donath S Improved techniques reduce face mask leak during simulated neonatal resuscitation: study 2. Arch Dis Child Fetal Neonatal Ed (2008) 93:F230–4. 10.1136/adc.2007.117788 18039750

[38] Schmölzer GM Roehr CC. Use of respiratory function monitors during simulated neonatal resuscitation. Klin Padiatr (2011) 223:261–6. 10.1055/s-0031-1275696 21630178

[39] Dold SK Schmölzer GM Kelm M Davis PG Schmalisch G Roehr CC. Training neonatal cardiopulmonary resuscitation: can it be improved by playing a musical prompt? A pilot study. Am J Perinatol (2014) 31:245–8. 10.1055/s-0033-1345261 23696429

[40] Roehr CC Schmölzer GM Thio M Dawson JA Dold SK Schmalisch G How ABBA may help improve neonatal resuscitation training: auditory prompts to enable coordination of manual inflations and chest compressions. J Paediatr Child Health (2014) 50:444–8. 10.1111/jpc.12507 24612106

[41] Schmölzer GM Morley CJ Wong C Dawson JA Kamlin CO Donath S Respiratory function monitor guidance of mask ventilation in the delivery room: a feasibility study. J Pediatr (2012) 160(3):377–81. 10.1016/j.jpeds.2011.09.017 22056350

[42] Hooper SB Fouras A Siew ML Wallace MJ Kitchen MJ te Pas A Expired CO2 levels indicate degree of lung aeration at birth. PLoS One (2013) 8(8):e70895. 10.1371/journal.pone.0070895 23951032 PMC3741323

[43] Mian Q Cheung PY O’Reilly M Pichler G van Os S Kushniruk K Spontaneously breathing preterm infants change in tidal volume to improve lung aeration immediately after birth. J Pediatr (2015) 167(2):274.e–8.e. 10.1016/j.jpeds.2015.04.047 25998060

[44] Hillman NH Kallapur SG Pillow JJ Moss TJM Polglase GR Nitsos I Airway injury from initiating ventilation in preterm sheep. Pediatr Res (2010) 67(1):60–5. 10.1203/PDR.0b013e3181c1b09e 19816239 PMC2795027

[45] Vento M Cheung PY Aguar M The first golden minutes of the extremely-low-gestational age neonate: a gentle approach. Neonatology (2009) 95:286–98. 10.1159/000178770 19052475

[46] O’Donnell CPF Davis PG Morley CJ. Resuscitation of premature infants: what are we doing wrong and can we do better? Biol Neonate (2003) 84:76–82. 10.1159/000071008 12890941

[47] Probyn ME Hooper SB Dargaville PA McCallion N Harding R Morley CJ. Effects of tidal volume and positive end-expiratory pressure during resuscitation of very premature lambs. Acta Paediatr (2005) 94(12):1764–70. 10.1080/0803525051002961 16421037

[48] Hernandez LA Peevy KJ Moise AA Parker JC. Chest wall restriction limits high airway pressure-induced lung injury in young rabbits. J Appl Physiol (1989) 66(5):2364–8. 2745302 10.1152/jappl.1989.66.5.2364

[49] Hillman NH Moss TJ Nitsos I Jobe AH. Moderate tidal volumes and oxygen exposure during initiation of ventilation in preterm fetal sheep. Pediatr Res (2012) 72(6):593–9. 10.1038/pr.2012.135 23037872 PMC4073615

[50] Schmölzer GM Kamlin OCOF O’Donnell CPF Dawson JA Morley CJ Davis PG. Assessment of tidal volume and gas leak during mask ventilation of preterm infants in the delivery room. Arch Dis Child Fetal Neonatal Ed (2010) 95(6):F393–7. 10.1136/adc.2009.174003 20547584

[51] Schmölzer GM Kamlin COF Dawson JA Morley CJ Davis PG. Tidal volume delivery during surfactant administration in the delivery room. Intensive Care Med (2011) 37:1833–9. 10.1007/s00134-011-2366-2 21976187

[52] Wallace MJ Probyn ME Zahra VA Crossley K Cole TJ Davis PG Early biomarkers and potential mediators of ventilation-induced lung injury in very preterm lambs. Respir Res (2009) 10:19. 10.1186/1465-9921-10-19 19284536 PMC2662809

[53] Björklund LJ Ingimarsson J Curstedt T John J Robertson B Werner O Manual ventilation with a few large breaths at birth compromises the therapeutic effect of subsequent surfactant replacement in immature lambs. Pediatr Res (1997) 42:348–55. 10.1203/00006450-199709000-00016 9284276

[54] Wada K Jobe AH Ikegami M. Tidal volume effects on surfactant treatment responses with the initiation of ventilation in preterm lambs. J Appl Physiol (1997) 83:1054–61. 9338410 10.1152/jappl.1997.83.4.1054

[55] Hillman NH Nitsos I Berry C Pillow JJ Kallapur SG Jobe AH Positive end-expiratory pressure and surfactant decrease lung injury during initiation of ventilation in fetal sheep. Am J Physiol Lung Cell Mol Physiol (2011) 301(5):L712–20. 10.1152/ajplung.00157.2011 21856815 PMC3290453

[56] Polglase GR Hillman NH Pillow JJ Cheah FC Nitsos I Moss TJM Positive end-expiratory pressure and tidal volume during initial ventilation of preterm lambs. Pediatr Res (2008) 64(5):517–22. 10.1203/PDR.0b013e3181841363 18596572 PMC2637939

[57] Ranieri VM Giunta F Suter PM Slutsky AS Mechanical ventilation as a mediator of multisystem organ failure in acute respiratory distress syndrome. JAMA (2000) 284(1):43–4. 10.1001/jama.284.1.43 10872010

[58] Bohrer B Silveira RC Neto EC Procianoy RS. Mechanical ventilation of newborns infant changes in plasma pro- and anti-inflammatory cytokines. J Pediatr (2010) 156(1):16–9. 10.1016/j.jpeds.2009.07.027 19783005

[59] Khwaja O Volpe JJ. Pathogenesis of cerebral white matter injury of prematurity. Arch Dis Child Fetal Neonatal Ed (2008) 93(2):F153–61. 10.1136/adc.2006.108837 18296574 PMC2569152

[60] Carvalho CG Silveira RC Procianoy RS. Ventilator-induced lung injury in preterm infants. Rev Bras Ter Intensiva (2013) 25(4):319–26. 10.5935/0103-507X.20130054 24553514 PMC4031878

[61] Dammann O Leviton A Bartels DB Dammann CEL Lung and brain damage in preterm newborns. Biol Neonate (2004) 85:305–13. 10.1159/000078175 15218288

[62] Walsh MC Morris BH Wrage LA Vohr BR Poole WK Tyson JE Extremely low birthweight neonates with protracted ventilation: mortality and 18-month neurodevelopmental outcomes. J Pediatr (2005) 146(6):798–804. 10.1016/j.jpeds.2005.01.047 15973322

[63] Polglase G Nitsos I Baburamani AA Crossley KJ Slater MK Gill AW Inflammation in utero exacerbates ventilation-induced brain injury in preterm lambs. J Appl Physiol (2012) 112:481–9. 10.1152/japplphysiol.00995.2011 22052871

[64] Mian Q Cheung PY Polglase G O’Reilly M Kushniruk K Aziz K Does high tidal volume delivery during positive pressure ventilation at birth cause brain injury in preterm infants? Proceedings of the Pediatric Academic Societies Annual Meeting (2015). 1594.707 p.

[65] Polglase G Hooper SB Gill AW Allison BJ McLean IN Pillow J Cardiovascular and pulmonary consequences of airway recruitment in preterm lambs. J Appl Physiol (2009) 106(4):1347–55. 10.1152/japplphysiol.91445.2008 19213936

[66] Polglase GR Moss TJM Nitsos I Allison BJ Pillow J Hooper SB. Differential effect of recruitment maneuvres on pulmonary blood flow and oxygenation during HFOV in preterm lambs. J Appl Physiol (2008) 105:603–10. 10.1152/japplphysiol.00041.2008 18535129

[67] Polglase GR Morley CJ Crossley KJ Dargaville P Harding R Morgan DL Positive end-expiratory pressure differentially alters pulmonary hemodynamics and oxygenation in ventilated, very premature lambs. J Appl Physiol (2005) 99(4):1453–61. 10.1152/japplphysiol.00055.2005 15890759

[68] Shekerdemian L Bohn D Cardiovascular effects of mechanical ventilation. Arch Dis Child (1999) 80(5):475–80. 10.1136/adc.80.5.475 10208959 PMC1717913

[69] Wong FY Leung TS Austin T Wilkinson M Meek JH Wyatt JS Impaired autoregulation in preterm infants identified by using spatially resolved spectroscopy. Pediatrics (2008) 121(3):e604–11. 10.1542/peds.2007-1487 18250118

[70] Greisen G. Autoregulation of cerebral blood flow in newborn babies. Early Hum Dev (2005) 81(5):423–8. 10.1016/j.earlhumdev.2005.03.005 15935919

[71] Soul JS Hammer PE Tsuji M Saul JP Bassan H Limperopoulos C Fluctuating pressure-passivity is common in the cerebral circulation of sick premature infants. Pediatr Res (2007) 61(4):467–73. 10.1203/pdr.0b013e31803237f6 17515873

[72] Threlkeld SW Lynch JL Lynch KM Sadowska GB Banks WA Stonestreet BS. Ovine proinflammatory cytokines cross the murine blood-brain barrier by a common saturable transport mechanism. Neuroimmunomodulation (2010) 17(6):405–10. 10.1159/000288265 20516722 PMC2914440

[73] Barton SK Melville JM Tolcos M Polglase GR McDougall ARA Azhan A Human amnion epithelial cells modulate ventilation-induced white matter pathology in preterm lambs. Dev Neurosci (2015) 37(4–5):338–48. 10.1159/000371415 25720586

[74] Yanowitz TD Jordan JA Gilmour CH Towbin R Bowen A Roberts JM Hemodynamic disturbances in premature infants born after chorioamnionitis: association with cord blood cytokine concentrations. Pediatr Res (2002) 51(3):310–6. 10.1203/00006450-200203000-00008 11861935

[75] Fotopoulos S Pavlou K Skouteli H Papassotiriou I Lipsou N Xanthou M. Early markers of brain damage in premature low-birth-weight neonates who suffered from perinatal asphyxia and/or infection. Biol Neonate (2001) 79:213–8. 10.1159/000047094 11275654

[76] Barton SK McDougall ARA Melville JM Moss TJ Zahra VA Lim T Differential short-term regional effects of early high dose erythropoietin on white matter in preterm lambs after mechanical ventilation. J Physiol (Lond) (2015). 10.1113/JP271376 26332509 PMC4771775

[77] Baik N Urlesberger B Schwaberger B Schmölzer GM Avian A Pichler G. Cerebral haemorrhage in preterm neonates: does cerebral regional oxygen saturation during the immediate transition matter? Arch Dis Child Fetal Neonatal Ed (2015) 100(5):F422–7. 10.1136/archdischild-2014-307590 26066762

[78] Stolp HB Liddelow SA Sá-Pereira I Dziegielewska KM Saunders NR. Immune responses at brain barriers and implications for brain development and neurological function in later life. Front Integr Neurosci (2013) 7:61. 10.3389/fnint.2013.00061 23986663 PMC3750212

[79] Schmölzer GM O’Reilly M Davis PG Cheung PY Roehr CC. Confirmation of correct tracheal tube placement in newborn infants. Resuscitation (2013) 84(6):731–7. 10.1016/j.resuscitation.2012.11.028 23211476

[80] Singh Y Oddie S. Marked variation in delivery room management in very preterm infants. Resuscitation (2013) 84(11):1558–61. 10.1016/j.resuscitation.2013.06.026 23948446 PMC3828483

[81] Morley CJ Davis PG Doyle LW Brion LP Hascoet JM Carlin JB. Nasal CPAP or intubation at birth for very preterm infants. N Engl J Med (2008) 358(7):700–8. 10.1056/NEJMoa072788 18272893

[82] Finer NN Carlo WA Walsh MC Rich W Gantz MG Laptook AR Early CPAP versus surfactant in extremely preterm infants. N Engl J Med (2010) 362(21):1970–9. 10.1056/NEJMoa0911783 20472939 PMC3071534

[83] Gittermann MK Fusch C Gittermann AR Regazzoni BM Moessinger AC. Early nasal continuous positive airway pressure treatment reduces the need for intubation in very low birth weight infants. Eur J Pediatr (1997) 156(5):384–8. 10.1007/s004310050620 9177982

[84] Dunn MS Kaempf J de Klerk A de Klerk R Reilly M Howard D Randomized trial comparing 3 approaches to the initial respiratory management of preterm neonates. Pediatrics (2011) 128(5):e1069–76. 10.1542/peds.2010-3848 22025591

[85] Tapia JL Urzua S Bancalari A Meritano J Torres G Fabres J Randomized trial of early bubble continuous positive airway pressure for very low birth weight infants. J Pediatr (2012) 161(1):75–80e1. 10.1016/j.jpeds.2011.12.054 22402568

[86] Stevens TP Finer NN Carlo WA Szilagyi PG Phelps DL Walsh MC Respiratory outcomes of the surfactant positive pressure and oximetry randomized trial (SUPPORT). J Pediatr (2014) 165(2):240.e–9.e. 10.1016/j.jpeds.2014.02.054 24725582 PMC4111960

[87] Vaucher YE Peralta-Carcelen M Finer NN Carlo WA Gantz MG Walsh MC Neurodevelopmental outcomes in the early CPAP and pulse oximetry trial. N Engl J Med (2012) 367(26):2495–504. 10.1056/NEJMoa1208506 23268664 PMC4140695

[88] Loeliger M Inder TE Cain S Ramesh RC Camm E Thomson MA Cerebral outcomes in a preterm baboon model of early versus delayed nasal continuous positive airway pressure. Pediatrics (2006) 118(4):1640–53. 10.1542/peds.2006-0653 17015557

[89] Dargaville PA Lavizzari A Padoin P Black D Zonneveld E Perkins E An authentic animal model of the very preterm infant on nasal continuous positive airway pressure. Intensive Care Med Exp (2015) 3:51. 10.1186/s40635-015-0051-4 26215815 PMC4512986

[90] Sobotka KS Hooper SB Allison BJ Te Pas AB Davis PG Morley CJ An initial sustained inflation improves the respiratory and cardiovascular transition at birth in preterm lambs. Pediatr Res (2011) 70(1):56–60. 10.1038/pr.2011.281 21659961

[91] te Pas AB Siew M Wallace MJ Kitchen MJ Fouras A Lewis RA Effect of sustained inflation length on establishing functional residual capacity at birth in ventilated premature rabbits. Pediatr Res (2009) 66(3):295–300. 10.1203/PDR.0b013e3181b1bca4 19542905

[92] Hillman NH Kemp MW Miura Y Kallapur SG Jobe AH. Sustained inflation at birth did not alter lung injury from mechanical ventilation in surfactant-treated fetal lambs. PLoS One (2014) 9(11):e113473. 10.1371/journal.pone.0113473 25419969 PMC4242618

[93] Sobotka KS Hooper SB Crossley KJ Ong T Schmölzer GM Barton SK Rapid cardiorespiratory recovery from severe asphyxia causes adverse cerebral haemodynamic changes and vascular leakage in near-term lambs. PLoS One (2015).

[94] Schmölzer GM Kumar M Aziz K Pichler G O’Reilly M Lista G Sustained inflation versus positive pressure ventilation at birth: a systematic review and meta-analysis. Arch Dis Child Fetal Neonatal Ed (2014) 100(4):F361–8. 10.1136/archdischild-2014-306836 25550472

[95] Lindner W Högel J Pohlandt F Sustained pressure-controlled inflation or intermittent mandatory ventilation in preterm infants in the delivery room? A randomised, controlled trial on initial respiratory support via nasopharyngeal tube. Acta Paediatr (2005) 94:303–9. 10.1080/08035250410023647 16028648

[96] te Pas AB Walther FJ. A randomized, controlled trial of delivery-room respiratory management in very preterm infants. Pediatrics (2007) 120(2):322–9. 10.1542/peds.2007-0114 17671058

[97] Lista G Boni L Scopesi F Mosca F Trevisanuto D Messner H Sustained lung inflation at birth for preterm infants: a randomized clinical trial. Pediatrics (2015) 135(2):e457–64. 10.1542/peds.2014-1692 25624390

[98] Chow SSW Report of the Australian and New Zealand Neonatal Network. Sydney, NSW: ANZNN (2011).

[99] Wilkinson D Andersen C O’Donnell CPF De Paoli AG. High flow nasal cannula for respiratory support in preterm infants. Cochrane Database Syst Rev (2011) (5):CD006405. 10.1002/14651858.CD006405.pub2 21563154

[100] Shoemaker MT Pierce MR Yoder BA DiGeronimo RJ. High flow nasal cannula versus nasal CPAP for neonatal respiratory disease: a retrospective study. J Perinatol (2007) 27(2):85–91. 10.1038/sj.jp.7211647 17262040

[101] Manley BJ Owen LS Doyle LW Andersen CC Cartwright DW Pritchard MA High-flow nasal cannulae in very preterm infants after extubation. N Engl J Med (2013) 369:1425–33. 10.1056/NEJMoa1300071 24106935

[102] Roberts CT Dawson JA Alquoka E Carew PJ Donath SM Davis PG Are high flow nasal cannulae noisier than bubble CPAP for preterm infants? Arch Dis Child Fetal Neonatal Ed (2014) 99(4):F291–5. 10.1136/archdischild-2013-305033 24625433

[103] Roberts CT Manley BJ Dawson JA Davis PG. Nursing perceptions of high-flow nasal cannulae treatment for very preterm infants. J Paediatr Child Health (2014) 50(10):806–10. 10.1111/jpc.12636 24943729

[104] Kugelman A Riskin A Said W Shoris I Mor F Bader D A randomized pilot study comparing heated humidified high-flow nasal cannulae with NIPPV for RDS. Pediatr Pulmonol (2015) 50(6):576–83. 10.1002/ppul.23022 24619945

